# Reliability of environmental DNA surveys to detect pond occupancy by newts at a national scale

**DOI:** 10.1038/s41598-022-05442-1

**Published:** 2022-01-25

**Authors:** Andrew Buxton, Alex Diana, Eleni Matechou, Jim Griffin, Richard A. Griffiths

**Affiliations:** 1 Durrell Institute of Conservation and Ecology, School of Anthropology and Conservation, University of Kent, Marlowe Building, Canterbury, Kent, CT2 7NR UK; 2 The Royal Agricultural University, Stroud Rd, Cirencester, GL7 6JS UK; 3 School of Mathematics, Statistics and Actuarial Science, University of Kent, Sibson Building, Canterbury, CT2 7FS UK; 4 Department of Statistical Science, University College London, 196-199 Tottenham Court Rd, Bloomsbury, London, W1T 7PJ UK

**Keywords:** Ecology, Ecological modelling, Molecular ecology, Wetlands ecology

## Abstract

The distribution assessment and monitoring of species is key to reliable environmental impact assessments and conservation interventions. Considerable effort is directed towards survey and monitoring of great crested newts (*Triturus cristatus*) in England. Surveys are increasingly undertaken using indirect methodologies, such as environmental DNA (eDNA). We used a large data set to estimate national pond occupancy rate, as well as false negative and false positive error rates, for commercial eDNA protocols. Additionally, we explored a range of habitat, landscape and climatic variables as predictors of pond occupancy. In England, 20% of ponds were estimated to be occupied by great crested newts. Pond sample collection error rates were estimated as 5.2% false negative and 1.5% false positive. Laboratory error indicated a negligible false negative rate when 12 qPCR replicates were used. Laboratory false positive error was estimated at 2% per qPCR replicate and is therefore exaggerated by high levels of laboratory replication. Including simple habitat suitability variables into the model revealed the importance of fish, plants and shading as predictors of newt presence. However, variables traditionally considered as important for newt presence may need more precise and consistent measurement if they are to be employed as reliable predictors in modelling exercises.

## Introduction

When undertaking any assessment of species distribution, it is imperative to understand the limitations of the survey methods used. Failure to fully consider sampling efficiency, sampling bias or other methodological limitations can lead to erroneous conclusions. Within nature conservation, these may result in inadequate assessments of impact, and inappropriate conservation target setting, leading to poor conservation outcomes^1–3^.

All survey methods suffer from imperfect detection, and indicators of species presence do not necessarily correspond to reality. At its most simplistic, a species may be observed if it is present (true positive) or not observed if it is not present (true negative). However, the species may not be observed even if it is present (false negative), or can be erroneously recorded when absent (false positive)^4–9^. Understanding the level of error for a particular survey method therefore improves confidence in any conclusions that emerge from downstream analysis.

Great crested newts (*Triturus cristatus*) are extensively surveyed within England, Wales and Scotland, as part of environmental impact assessments, and to monitor trends in conservation status. The high degree of conservation effort dedicated to this species is a direct result of its listing within several legal instruments, including Schedule 5 of the Wildlife and Countryside Act 1981 (as amended) and Schedule 2 of the Conservation of Habitats and Species (Amendment) (EU Exit) Regulations 2019. Indeed, the tendency for the preferred habitat for the species to overlap with areas subject to development pressure is a further driver of intense survey effort^10^. Until recently, surveys of great crested newts have focused on direct observations using traps, nocturnal visual encounter surveys, pond nets and searching for eggs on vegetation^11,12^. To maximise detectability and reduce the risk of false negatives, up to seven survey visits using a combination of methods are required^13^. More recently, surveys targeting environmental DNA (eDNA) have become available and have been added to the suite of available methods^14^. Surveys targeting eDNA can be conducted very rapidly, with a surveyor able to visit multiple sites in one day. Indeed, the use of eDNA has allowed for national distribution assessments of the species^15^ to be conducted on temporal and spatial scales that would have been prohibitive using other methods.

Site occupancy modelling requires repeat visits to the same sites to account for imperfect detection, ascertain the likelihood of observing a species if it is present (detection probability) and estimate the proportion of sites that are occupied (occupancy probability), with provisions to take into account covariates^16–19^. However, standard occupancy models assume that false positive observations do not occur. This assumption is not always met, particularly in cases where a species may be misidentified^20^ or in cases of indirect survey methods where the species is inferred to be present through biological products (e.g. tracks, faeces, hair). eDNA false positive error may not be negligible in surveys^7^ and—as with all methods—needs to be accounted for when assessing site occupancy. Surveys targeting eDNA may incur such imperfect detection at two levels—sample collection in the field and sample analysis in the laboratory. Recently, models have been developed that allow for multiscale occupancy models to be applied, which account for both positive and negative error^21^ at both stages.

The ability to undertake a rapid national distribution assessment using eDNA surveys provides opportunities for data exploration that are not possible using traditional methods. Firstly, using observations from thousands of sites permits reliable and large-scale estimates of site occupancy. Secondly, it provides opportunities to explore how occupancy and detection rates are influenced by habitat covariates. Although occupancy modelling has been previously applied to great crested newt eDNA data, the examples have used either localised data^22^, analysis using standard occupancy models^22^ or have been presented in the literature in a methodological or model development context^21,23,24^. As a result, outputs from national data sets have yet to be examined in an ecological context that relate to great crested newt eDNA surveys.

Habitat covariates are frequently collected within the standard great crested newt Habitat Suitability Index (HSI) originally developed by Oldham et al.^25^ and updated within ARG UK^26^. The great crested newt HSI is a rapid and very simple habitat quality assessment for the species that considers ten variables, assigning a value of between 0.01 and 1 to each, and derives an overall HSI by taking the geometric mean of these values. This approach gives each covariate equal weighting within the analysis, an assumption that is unlikely to be met in practice.

Using an eDNA data set collected from across England, we use the Griffin et al. (2020) model^21^ via the eDNAShinyApp package^23^, within statistical software R^27^, to examine pond occupancy and false positive and false negative error rates of surveys targeting great crested newt eDNA. This allows us to estimate national pond great crested newt occupancy rates and to consider habitat covariates collected alongside the eDNA samples, or gleaned from location data, to relate pond occupancy to site characteristics.

## Results

A threshold of a single positive qPCR replicate is typically applied for assigning site occupancy with eDNA surveys^14^, however; in some studies, samples with low numbers of qPCR replicates amplifying have been reanalysed, in an effort to reduce false positive error^28^. An observed (naïve) pond occupancy rate of 0.30 (1496 occupied sites) was obtained for the data based on a threshold of a single qPCR replicate amplifying. This was reduced to 0.25 (1237 occupied sites) when a threshold of two amplifying qPCR replicates was applied and 0.22 (1097 occupied site) when the threshold was increased to three.

The mean of the posterior mean occupancy for all sites was 0.198 with 95 percentiles of 0.02 and 0.50 respectively. The false positive rate was found to be very low, both at Stage 1, sample collection ($\theta_{10}$: 0.015; PCI = 0.0005 to 0.035), and Stage 2, laboratory analysis ($p_{10}$: 0.020; PCI = 0.018 to 0.022), while the true positive rate was high at both Stage 1 ($\theta_{11}$: 0.948; PCI = 0.749 to 0.999) and Stage 2 ($p_{11}$: 0.808; PCI = 0.799 to 0.818). False negative probability is calculated as 1—true positive: this was higher than the false positive rate at both Stage 1 (0.052), and Stage 2 (0.192).

**Figure 1:**
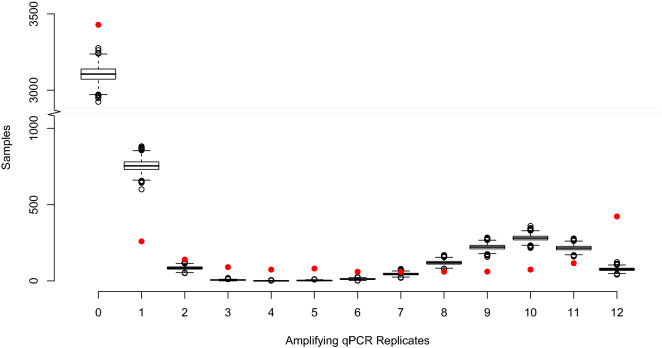
Frequency of qPCR replicate response within the observed data set (red dots), with boxplots representing the simulated median, IQR and whiskers at quartile + /− 1.5 IQR for the 100 repeats of simulated data, based on the modelled individual site occupancy and probabilities for both Stage 1 and Stage 2 true and false positives from the goodness of fit analysis.

The observed amplifying qPCR replicate frequency showed high numbers of samples between zero and two as well as at complete or nearly complete amplification (11 or 12 of the 12 replicates amplifying: Fig. 1). A similar pattern was observed in data simulated from the goodness of fit model; however, our observed data suggested greater numbers of both no amplification, and full amplification samples than expected by the model. We observed higher numbers of samples amplifying with two to six replicates, than predicted by the simulations: the median number of samples expected to amplify between three and six qPCR replicates was just six, zero, two and twelve respectively. In comparison, we observed lower numbers amplifying with one, eight, nine, ten and eleven replicates, with a peak in expected amplification at ten replicates (Fig. 1).

**Figure 2:**
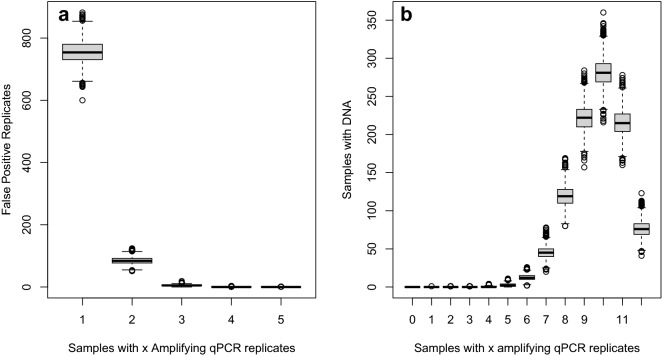
Goodness of fit summary of the number of (**a**) simulated false positives qPCR replicates in a sample, when DNA is not present, at Stage 2 and (**b**) simulated number of positive qPCRs for samples with DNA present at Stage 2. 0 represents samples where all qPCR replicates are incorrect, while 12 represents samples where all qPCR replicates are correct.

**Figure 3:**
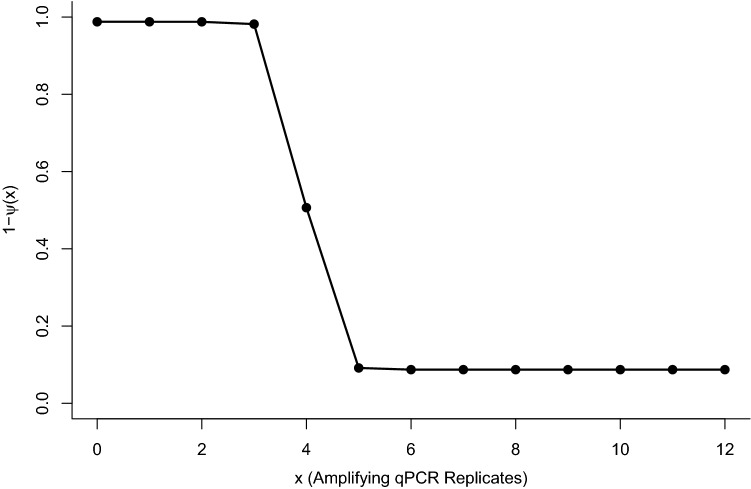
The posterior conditional probabilities of species absence (1—ψ (x)), given x amplifying qPCR replicates.

We examined the number of false positive qPCR replicates at Stage 2 (i.e., the number of amplifying qPCR replicates when DNA is not present) under the fitted model. Even though each replicate was only found to have a probability of 0.02 of being a false positive, the high levels of replication mean this is magnified at the sample level. With 12 qPCR replicates at Stage 2, the probability of at least one replicate returning a false positive was estimated to be 0.24. We compared the median number of samples expected to amplify at different levels of qPCR replication with the median number expected to be false positive at Stage 2. Under the fitted model, all simulated samples amplifying with one or two qPCR replicates are false (Figs. 1,2a). However, instances where four or more Stage 2 false positive qPCR replicates were obtained for a single sample were very rare. This concurs with the conditional probability of species absence analysis, which identified a high probability of species absence when between 0 and 3 replicates amplified, dropping through four, then a low probability of species absence for samples that amplified with between 5 and 12 replicates (Fig. 3). We also examined the distribution of false negative qPCR replicates at Stage 2 under the fitted model (Fig. 2b). Although we see relatively high instances of between six and twelve qPCR replicates correctly amplifying when DNA is within a sample, the number of instances where fewer than five of the qPCR replicates amplified when DNA was present was very low.

**Figure 4:**
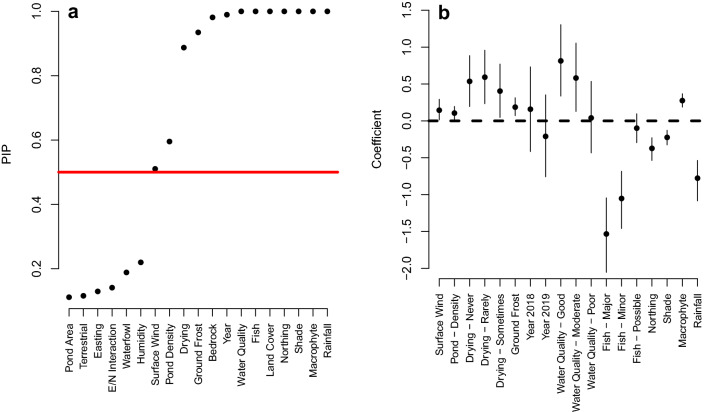
(**a**) PIP values for each covariate, those scoring above 0.5 (red line) were considered important predictors for great crested newt occupancy; and (**b**) occupancy coefficients and posterior credible intervals for each level for covariates, where PIP values were greater than 0.5. Land cover and geology have been omitted, for visual reasons, due to the large number of categories in each, these can be found in Table S1.

Out of the 19 covariates included within the model, 13 were considered to be important predictors of great crested newt pond occupancy. Water quality, shade, rainfall, Northing, macrophyte cover, land cover type, fish, survey year, bedrock, ground frost, frequency of drying, wind speed and pond density were all found to have Posterior Inclusion Probability (PIP) scores of greater than 0.5 for occupancy (Fig. 4a; Table S1), suggesting an influence on great crested newt pond occupancy. However, the credible intervals crossed zero for all levels of survey year and bedrock when compared to the intercept, (Fig. 4b); this was not the case when all categorical covariate level pairs were examined. A higher occupancy was identified in 2018 compared to 2019 (Table S2). Similarly, credible intervals did not cross zero for 27 of the over 500 possible bedrock type pair combinations, indicating that some rock types are important predictors of occupancy. In general, igneous and limestone outperformed sandstone and mudstone rock types, while mudstone outperformed sandstone rock types (Table S3). However, the individual influences are difficult to interpret due the number of categories as well as unbalanced, sometimes very small sample sizes. Terrestrial habitat, waterfowl intensity, pond area, humidity, Easting and the interaction between Easting and Northing all had PIP values below 0.5 and as a result were not revealed as important.

In ecological terms, improved water quality, a low but occasional frequency of drying, increased macrophyte cover, increased pond density, higher number of ground frost days and wind speed were linked with higher probability of pond occupancy. In contrast, increases in shade cover, fish intensity, rainfall, and more northerly latitudes were associated with lower probabilities of pond occupancy (Fig. 4a,b).

Landcover type (17 categorical levels included in the model) was also found to be an important predictor of pond occupancy (PIP = 1). Acid grassland was taken as the baseline level, with every other landcover type showing reduced occupancy compared to this level (Table S1). When all pairs of landcover type were examined, improved grassland and broad-leaved woodland showed an increase in suitability over arable farmland, while improved grassland also demonstrated higher occupancy rates than the freshwater category made up of rivers and lakes (Table S4, Figs. S1, S2).

## Discussion

Various estimates for great crested newt pond occupancy rates have been published with most relating to site or regional scale assessments. A naïve occupancy rate of 0.13 has been identified for a data set from the northwest of England^29^, while estimates based on conventional occupancy^16^ modelling of 0.31 for southeast England and between 0.32 and 0.33 for mid Wales were presented by Sewell et al.^13^. The only other national data which the authors are aware of are within the Freshwater Habitat National PondNet Study, which estimates a naïve pond occupancy of between 13 and 18%^30^, and the Amphibian and Reptile Conservation Trust National Amphibian and Reptile Recording Scheme, which suggests a 12% occupancy rate for the UK^31^. Using data from nearly 5000 ponds sampled across England, here we provide a more extensive national-level analysis while accounting for imperfect detection in the eDNA sampling protocol. Assuming a threshold of just one positive qPCR replicate in a sample, the naïve occupancy estimate of 0.30 is similar to the localised regional estimates made by Sewell et al.^13^ using direct observation methods. The posterior mean estimates of 0.198 for occupancy are comparable to most other estimates for great crested newt pond occupancy in the UK, but lower than the naïve estimate. The lower modelled estimates of occupancy than the naïve estimate suggest that false positives should not be ignored and need to be accounted for statistically using methodologies such as the eDNAShinyApp package used here^23,24,27^.

The goodness of fit analysis was based on the MCMC output for each site and observed covariate levels in the data set. Some lack of fit was observed, with a predicted peak in amplification at 10 qPCR replicates but an observed peak at 12 qPCR replicates. There are several potential causes for this. For example, variation between laboratories could not be accounted for as these metadata were not made available. The assumption that error rates are the same across all laboratories may therefore not apply and could contribute to poorer model fit. Secondly, we did not consider error rates as functions of covariates, and this may also have contributed to a poorer fit.

Stage 1 error was found to be smaller than Stage 2 error for both false positive and false negative error. However, Stage 2 error operates on individual qPCR replicates and not at the site level. If there was no error at Stage 2, we would observe either zero qPCR replicates amplifying or all qPCR replicates amplifying (i.e. 12 in the case of the data presented here). The majority of samples showed zero qPCR amplification (3429 samples), and this was strongly linked to absence of newts. For sites with amplification, we observed a greater number of samples amplifying between 1 and 11 qPCR replicates (1074 samples) than we did amplifying with all 12 qPCR replicates (422 samples). The qPCR replicates that do not amplify in samples containing target DNA are erroneous, even if other replicates within that sample do amplify and contribute to this high Stage 2 false negative error in the model output. Data simulated from the fitted model show that the frequency of samples that contain DNA at Stage 2 amplifying in less than five of the 12 qPCR replicates is very low (Fig. 2b). Given that all replicates need to be erroneous to alter the naïve assignment of a sample containing DNA to negative, Stage 2 false negatives at this sampling level are unlikely. However, this does not rule out Stage 1 false negative error which we estimate to be 5.2% (with wide credible intervals between 0.1% and 25.1%).

Higher levels of Stage 2 replication remove lab-based false negative error. If eDNA is present within a sample and a high number of replicates are used, it is highly unlikely that all qPCR replicates will be erroneously negative, even when the false negative rate at the replicate level is high. Conversely, high levels of Stage 2 replication increase the likelihood of false positive error occurring^32^. Stage 2 false positive results are of greater consequence than the 2% the model output would suggest. Unlike false negative error where all Stage 2 replicates need to be erroneous to change the naïve assignment of occupancy of a sample, when a threshold of one amplifying replicate is applied, only a single replicate needs to be an erroneous to generate a false positive. With 12 qPCR replicates at Stage 2 and a 2% false positive error per replicate, a sample with no DNA present has a 24% chance of producing at least one amplification. Assuming this error is randomly distributed through samples with no DNA present and qPCR replicates, it is more likely that samples with small numbers of replicates amplifying would be erroneous than where large numbers of replicates amplify. This was confirmed in the goodness of fit analysis with the distribution of Stage 2 false positive replicates making up all samples amplifying with one or two positive qPCR replicates, while negligible false positive amplification was seen with four amplifying replicates or above (Fig. 2a). With only a single sample at Stage 1, false positive error is limited to the 1.5% per sample, as per the ${\theta }_{10}$ value in the occupancy model output.

We would recommend that, where possible, results from individual sites are interpreted as a probability of site occupancy, based on modelled outputs such as those produced by the eDNAShinyApp R package^23,27^. The precision of these models is dependent on sample size. Where sample size is large, a reduced bias and narrower credible interval range is observed^24^. However, using occupancy modelling, Buxton et al.^24^ demonstrated that studies that contain only a small number of sites are unlikely to produce accurate and precise estimates. As a result, such assessments will need to continue to rely on a threshold value of amplifying qPCR replicates to define site occupancy. A naïve amplification threshold for assigning occupancy of one positive qPCR replicate is unwise and should be increased to reduce Stage 2 false positive error. Indeed, a threshold of three positive qPCR replicates would reduce false positive error, without increasing false negative error. Alternatively, redistributing the replication between Stage 1 and Stage 2^24^, would also reduce the credible interval width and generate a more precise posterior mean estimate at Stage 1, in turn reducing the uncertainty around the occupancy estimate. A redistribution of replication leading to two samples collected from each site, both analysed using up to six qPCR replicates, as opposed to one sample analysed using twelve qPCR replicates, has been suggested^24^.

Equal weighting of the ten covariates used in the traditional great crested newt HSI assessment^25^ may be ecologically unrealistic^29^. This is supported by the observations here, with only some of the HSI covariates identified as important for occupancy. The model applied by the eDNAShinyApp package^23,27^ successfully identified several covariates known to influence great crested newt occupancy, that are included within the HSI assessment^25^. These included occurrence of fish, water quality, shade, pond density, macrophyte cover, frequency of drying and geographic area; although our analysis was based on Easting and Northing, rather than the broad-scale suitability map used in deriving the original HSI^25^. However, several traditionally used HSI variables emerged as unimportant, i.e., waterfowl, terrestrial habitat quality, and area of pond; while ground frost, rainfall, surface wind and land cover type, are not included within the HSI assessment but were important.

The importance and influence of the HSI suitability indices of fish, shade, pond density, water quality, macrophyte cover, and frequency of drying on pond occupancy were all as expected with wide literature support^25,33–48^. The negative pond occupancy response to climatic covariates of ground frost and high precipitation are supported in relation to annual survival^47^. ‘It is worth noting that the PIP value for wind speed was only just over the threshold for inclusion as important. Although ponds are shallow with limited stratification possible, wind speed has been shown to influence the distribution of eDNA in deeper waterbodies^49,50^. Estimating the presence of fish in a pond by direct observation for the traditional HSI may be problematical, and metabarcoding approaches to eDNA surveys which offer information on presence of other species would improve the accuracy of covariates, such as fish presence^40^. Indeed, assigning “Possible” fish presence within the HSI when scoring a pond accounted for the same percentage (33.1%) of both positive and negative eDNA samples. This suggests that when surveyors are not confident of fish presence, they are using this category in equal proportions for both occupied and unoccupied ponds. Landcover and bedrock were also important for pond level occupancy. This is expected given the importance of terrestrial habitat, and water retention to the species (Figs. S1, S3)^35,51^. However, with very unbalanced sample sizes between the categories (Figs. S2, S4), and influence of nearby land cover types uncaptured by the data, this variable is difficult to interpret, and we suggest further examination. Nevertheless, the positive associations with woodland and grassland reflect established knowledge of habitat preferences^36^. Equally, as freshwater predominantly relates to rivers and lakes rather than ponds in the landcover dataset used, negative relationships reflect the lower suitability of these habitats^36^.

Several covariates, however, did not exhibit the expected response for pond occupancy. Terrestrial habitat was not found to be important despite the species being only semi-aquatic, and previous studies emphasising the importance of this variable^36^. This may be a result of the original Oldham et al.^25^ terrestrial habitat assessment being simplified into four subjective categories in the ARG UK^26^ protocol: this may not be nuanced enough to differentiate terrestrial habitat usage using statistical modelling. Waterfowl were not identified by the model as important predictors of great crested newt pond occupancy, where they have been elsewhere^29,41^, with one study suggesting a positive relationship between waterfowl species richness and great crested newt occupancy^40^. The lack of importance demonstrated in this data set may indicate that other covariates outweigh waterfowl in terms of occupancy importance, or they may only become important predictors of occupancy at very high waterfowl densities rarely observed in this data set. Similarly, pond area was not found to be an important predictor of pond occupancy. There was no difference in the mean area for occupied or unoccupied ponds; however, no occupied ponds were found above 10,000 m^2^. We would anticipate that both very small and very large ponds to be unsuitable for great crested newts^25,52^.

Northing but not Easting was found to be an important predictor of pond occupancy. A distribution gradient with latitude is a common feature of biodiversity generally, and in the UK great crested newts are much more patchily distributed in Scotland than in England^53,54^. Pond occupancy estimates varied by year, with a greater occupancy in 2018 than the other years considered. This is likely linked to climatic conditions and may relate to the timings of ponds drying in relation to eDNA sample collection. This may therefore be an artefact of unoccupied ponds being more likely to dry early in the season and therefore being excluded from occupancy estimates for dry years, or local migration to less suitable habitat if core ponds start to dry, however long term analysis of individuals within a metapopulation shows little support for this^47^. As a result, in very dry years, we would expect an increase in pond occupancy to be observed in the data. Although average early spring rainfall for England in 2018 was higher than in either 2017 or 2019, rainfall during the main eDNA survey window of May and June was considerably less in 2018 than in the other two years (Fig. S5). Similar variation in year on year occupancy rate has been observed elsewhere^30^.

As with all sampling methods, imperfect detection is a general feature of eDNA surveys. When high levels of qPCR replicates are used, false negative error is predominantly due to failure to collect DNA in a sample rather than failure to detect DNA within the lab. False positive error can occur at both stages and is exaggerated at Stage 2 by high levels of replication; Stage 2 false positive error is most likely in samples with a low proportion of replicates amplifying. We recommend using statistical models to estimate the occupancy of individual sites, taking into consideration sampling error. Failing that, a naïve occupancy threshold of two or three amplifying qPCR replicates, adjusting for total levels of replication, should be applied before assigning a site as occupied or not.

With specific reference to great crested newts, we estimate approximately 20% of ponds through their natural range within England are occupied. We estimate that eDNA sampling failed to collect DNA from approximately 5% of sites where it was present. However, if eDNA is collected it is highly unlikely to be missed during the laboratory phase using the present protocol. We estimate that eDNA is erroneously collected in approximately 1.5% of water samples causing Stage 1 false positive results. However, false positives at the laboratory phase were found to be 2% per qPCR replicate; it is likely that this error would account for the majority of samples amplifying with one or two qPCR replicates, as a result these need to be treated with caution. To maximise accuracy, we recommend redistributing replication between the two stages, as is recommended elsewhere, and that thresholds to define a replicate as positive are further examined^24,55^. It is important to recognise that visual surveys also experience imperfect detection^13^, with observation errors likely to be similar to or greater than the error experienced using eDNA methods, particularly if the recommendations presented here are put in place to minimise laboratory stage false positive error. The benefits associated with eDNA over traditional methods allowing rapid collection of large scale distribution data are invaluable and should not be devalued in relation to traditional methods^15^. Although not identified within the models as important predictors, waterfowl, terrestrial habitat, and pond area may remain important habitat features for great crested newts. These covariates may be less important than the other HSI covariates, may not be measured in a sufficiently nuanced way to enable their importance to be identified, or may have influence on a local but not national scale^29,40^. However, equal weighing of the ten HSI variables is an oversimplification with the effect of some variables, for example pond area, overinflated within the HSI analysis, whereas others are undervalued, for example fish intensity. It is important to measure HSI covariates accurately and consistently to allow them to be utilised in statistical analysis such as this, and a review of the covariates and weighting is warranted now large occupancy data sets are becoming available.

## Methods

### eDNA data and study area

As part of a national distribution assessment for great crested newts, Natural England, the statutory agency responsible for nature conservation within England, commissioned widespread eDNA surveys, making the data publicly available on the Natural England Open Data Portal (see “Data availability”). eDNA surveys were undertaken following the standard commercially accepted methodology for great crested newt eDNA survey within the UK based on Biggs et al.^56^. These surveys consist of a composite sample of pond water collected from 20 locations around the perimeter of a pond, which is then combined before subsampling to fill six 50 ml centrifuge tubes that also contain 33 ml of absolute ethanol and 1.5 ml of 3 M sodium acetate solution, to aid in DNA preservation and precipitation. This results in a total sample volume of approximately 90 ml of pond water^14^. The samples are extracted via ethanol precipitation and a modified QIAGEN DNeasy Blood and Tissue Kit extraction protocol (see Biggs et al.^56^). Samples are analysed via qPCR, using primers and a hydrolysis probe^57^, with conditions and internal positive controls, described in Biggs et al.^56^. Twelve qPCR replicates were performed on each sample, with a replicate assigned as positive if an exponential growth phase was observed in the qPCR amplification curve, even if the Ct value was very high, as per the commercially accepted protocol^14^. In total, 5866 ponds were surveyed across England between 2017 and 2019. This was reduced to a sample of 4925 ponds after deletion of (1) samples that returned an inconclusive result (i.e., showed evidence of DNA degradation or PCR inhibition); (2) sites where associated covariate information was incomplete; and (3) sites where the geographic location was masked at a lower resolution than 100 m (see supplementary Fig. 6). The sample collection was undertaken by an unknown number of individuals with samples analysed at several laboratories.

### Covariates

Alongside each eDNA sample, an HSI assessment was undertaken by the commissioned survey teams^25^. We examined nine of the ten individual suitability indices (SIs), as described in the ARG UK advice note^26^: geographic location, pond area, frequency of pond drying, percentage of shoreline shading, water quality based on an invertebrate assessment, intensity of waterfowl use, intensity of fish use, density of ponds within 1 km, suitability of terrestrial habitat, and percentage of macrophyte cover^25,26^. We replaced the covariate comprising three broad geographic areas with more precise Easting and Northing values for individual pond locations. We considered the main effects are well as the interaction of Easting and Northing. We supplemented the HSI covariates with data from a number of additional sources. This included the year in which the eDNA sample was collected, together with the land cover type from the UKCEH Land Cover Maps 2019^58,59^ accessed via Digimap and the bedrock type as found on the BGS 1:650,000 geology layer^60^ also accessed via Digimap, was attached to each data point based on Easting and Northing. Additionally, we attached the HadUK-Grid Gridded Climate Observations in a 1 km grid to each data point, including: mean daily air temperature; mean daily minimum air temperature; mean daily maximum temperature; mean annual precipitation; mean annual sunshine hours; mean wind speed at 10 m; mean sea level pressure; mean vapour pressure; mean relative humidity; mean annual days with ground frost; and mean annual days with snow lying, each calculated over the 20 year period 2000 to 2019 inclusive^61^.

We calculated Pearson’s correlation coefficient for all pairs of continuous covariates using the cor function in base R version 4.0.5^27^. A correlation coefficient threshold of 0.7 was applied to define collinearity between pairs of covariates^62^. Where covariates were strongly correlated with one another, only one of a correlated pair was retained in the analysis. Covariates included within the model, following the removal of variables that were correlated with at least one of the existing variables, were year of survey, frequency of drying, water quality for amphibians, waterfowl, occurrence of fish, terrestrial habitat quality for amphibians, land cover type, bedrock type, Easting, Northing, pond area, perimeter shading, pond density, macrophyte cover, mean annual days with ground frost, mean relative humidity, mean annual precipitation, mean wind speed at 10 m and the interaction between Easting and Northing. Within the remaining covariates, the maximum correlation coefficient was found to be 0.52, between mean wind speed at 10 m and mean annual precipitation.

### The App

The R package eDNAShinyApp^23^ was run in R version 4.0.5^27^. The probabilities of occupancy ($\psi$), Stage 1 (sample collection) true positive ($\theta_{11}$) and false positive ($\theta_{10}$) observations and Stage 2 (laboratory analysis) true positive ($p_{11}$) and false positive ($p_{10}$) observations, were all considered with default prior settings from Griffin et al.^21^ of 0.9 for $\theta_{11}$ and $p_{11}$ and 0.1 for $\theta_{10}$ and $p_{10}$; however, only ψ was considered as a function of covariates. The package was run using 5,000 burn-in iterations, 3,000 iterations, 1 chain and 100 thinned iterations, with ‘probability of site occupancy’ set to 0.5, ‘variance of probability of site occupancy’ set to 4, ‘variance of coefficients of probability of site occupancy’ set to 0.25, number of significant covariates’ set to 2.

### Covariate importance selection

Bayesian variable selection using an Add-Delete-Swap approach is automated within the eDNAShinyApp package using a Pólya-Gamma sampling scheme and the Markov Chain Monte Carlo (MCMC) algorithm presented in Griffin et al.^21^. Covariates were considered important if their respective posterior inclusion probability (PIP) value was greater than 0.5, indicating that they appear in more than 50% of model iterations and the 95% posterior credible intervals of their corresponding coefficients did not include zero. High PIP values for categorical covariates but with corresponding 95% posterior credible interval (PCI) of all coefficients including zero suggest that there is no difference between any of the levels and the baseline level, but there is likely to be between other pairs of levels. In these cases, we considered posterior summaries, including 95% PCIs of the differences between all pairs of levels. Where these 95% PCIs did not cross zero, an important difference was identified between that pair of covariate levels.

### Goodness of Fit

We used the MCMC values obtained for all parameters to simulate the number of amplifying qPCR replicates for all S = 4925 sites, using M = 1 sample from each site and K = 12 PCR replicates for each sample. We repeated the simulations 100 times. This also generated the number of both true and false positive samples at Stage 1 and number of both true and false positive qPCR replicates at Stage 2, allowing us to quantify the level of error expected under the model.

## Supplementary Information

Supplementary Information 1.

Supplementary Information 2.

## Data Availability

The raw data used in the model comparison case study was collected by Natural England and is available through the Natural England Open Data Portal https://naturalengland-defra.opendata.arcgis.com/datasets/ffba3805a4d9439c95351ef7f26ab33c_0/data. The code to run the app is available here https://blogs.kent.ac.uk/edna/, while the code to simulate expected amplification distributions will be uploaded to Kent Academic Repository on acceptance of the manuscript. While the base software R is available here https://www.r-project.org/. Geological Map Data BGS UKRI 2021, available under licence from Digimap.
