# Supplementary material for: Reliability of environmental DNA surveys to detect pond occupancy by newts at a national scale
Source: Sci Rep. 2022 Jan 25;12:1295. doi: 10.1038/s41598-022-05442-1 (PMC8789902; doi:10.1038/s41598-022-05442-1)
Supplement: Supplementary file 1 — Supplementary Information 1. [file 41598_2022_5442_MOESM1_ESM.docx]

Reliability of environmental DNA surveys to detect pond occupancy by newts at a national scale

Authors:

Andrew Buxton* - Durrell Institute of Conservation and Ecology, School of Anthropology and Conservation, University of Kent, Marlowe Building, Canterbury, Kent, CT2 7NR, UK

andrews.buxton@btinternet.com

Alex Diana - School of Mathematics, Statistics and Actuarial Science, University of Kent, Sibson Building, Canterbury, CT2 7FS, UK

Eleni Matechou - School of Mathematics, Statistics and Actuarial Science, University of Kent, Sibson Building, Canterbury, CT2 7FS, UK

Jim Griffin – Department of Statistical Science, University College London, 196-199 Tottenham Court Rd, Bloomsbury, London, W1T 7PJ, UK

Richard A. Griffiths - Durrell Institute of Conservation and Ecology, School of Anthropology and Conservation, University of Kent, Marlowe Building, Canterbury, Kent, CT2 7NR, UK

* Corresponding Author

# **Supplementary Information:**

**Supplementary Tables Headings**

Table S1 – Covariates associated with great crested newt pond occupancy. PIP values greater than 0.5 indicate importance. Posterior mean and credible intervals for each variable and categorical level indicate the degree of the influence on occupancy compared to the intercept. If the PIP value is less than 0.5 the variable is considered to be unimportant.

Table S2 – Posterior mean and median values with 95% credible intervals for non-intercept level pairs of for the year categorical covariate. Pairings where credible intervals do not cross zero and are therefore important are highlighted in bold.

Table S3 – Matrix table showing the posterior mean and median values with 95% credible intervals for non-intercept level pairs of for the bedrock categorical covariate. Pairings where credible intervals do not cross zero and are therefore important are highlighted in bold.

Table S4 - Matrix table showing the posterior mean and median with 95% credible intervals for non-intercept level pairs of for the landcover categorical covariate. Pairings where credible intervals do not cross zero and are therefore important are highlighted in bold.

**Supplementary Graphs
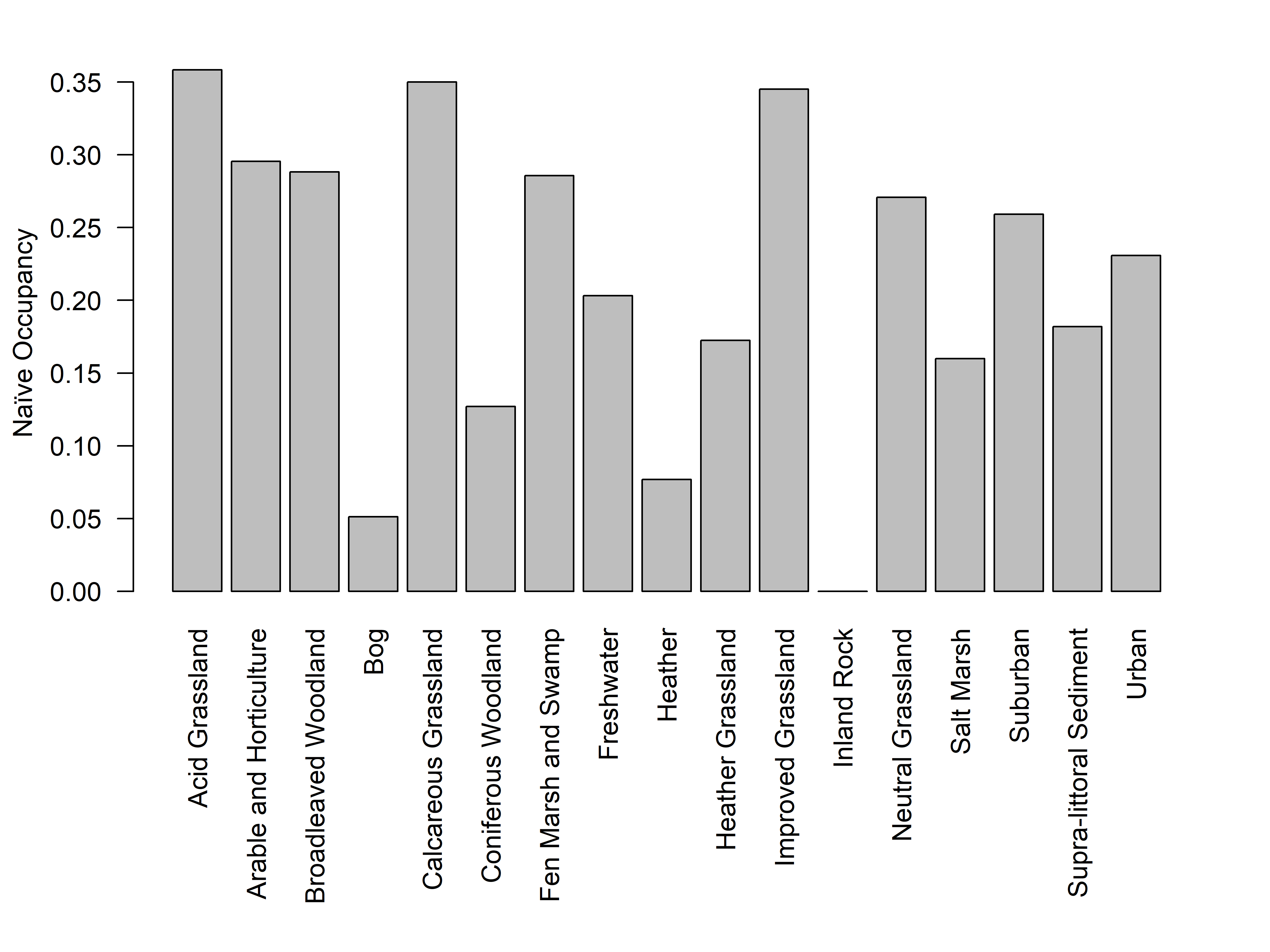
**

Figure S1 – Naïve great crested newt pond occupancy rate for each land cover type


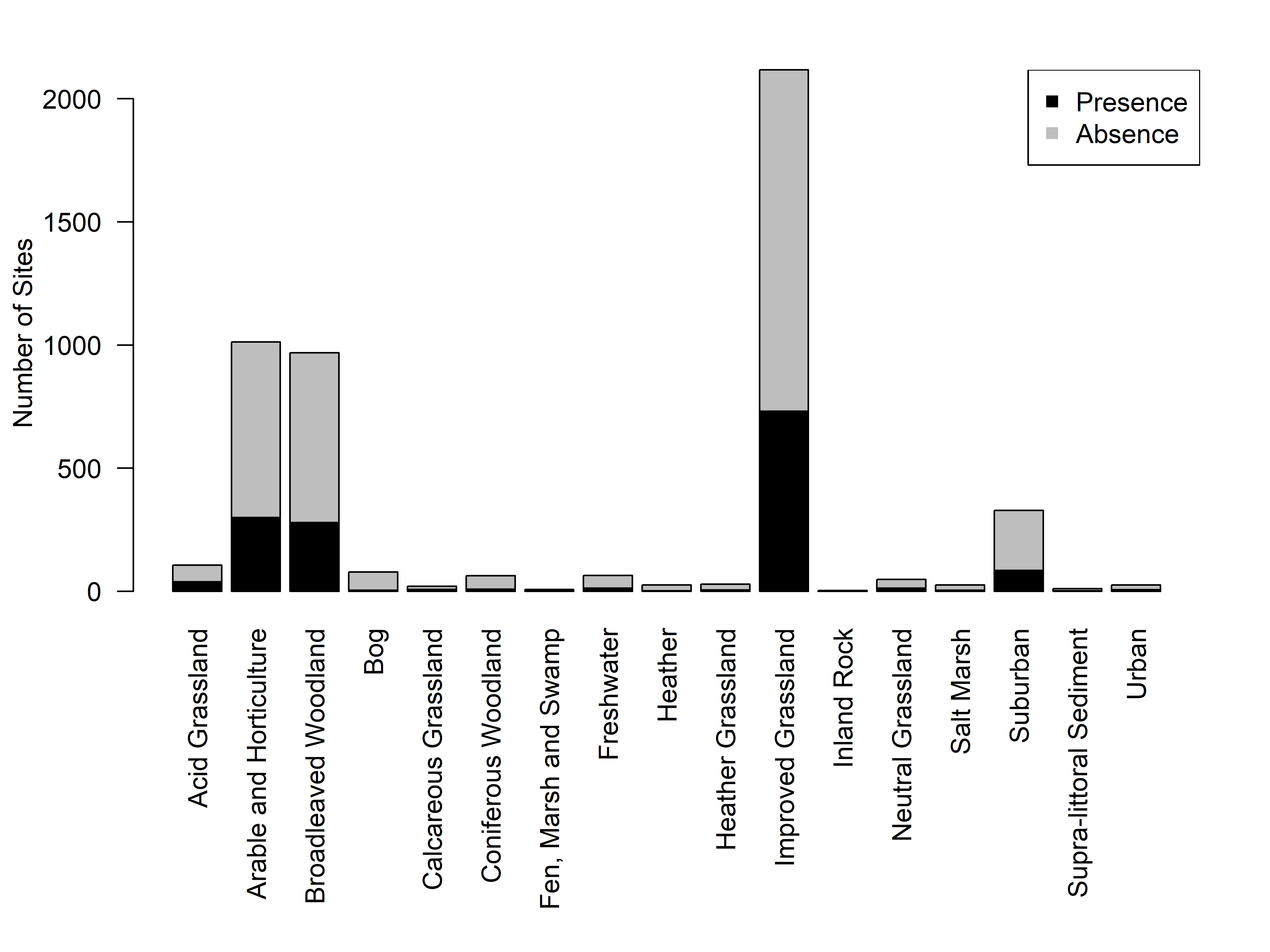


Figure S2 – the cumulative number of eDNA samples collected from each land cover type, with the great crested newt presence absence split shown


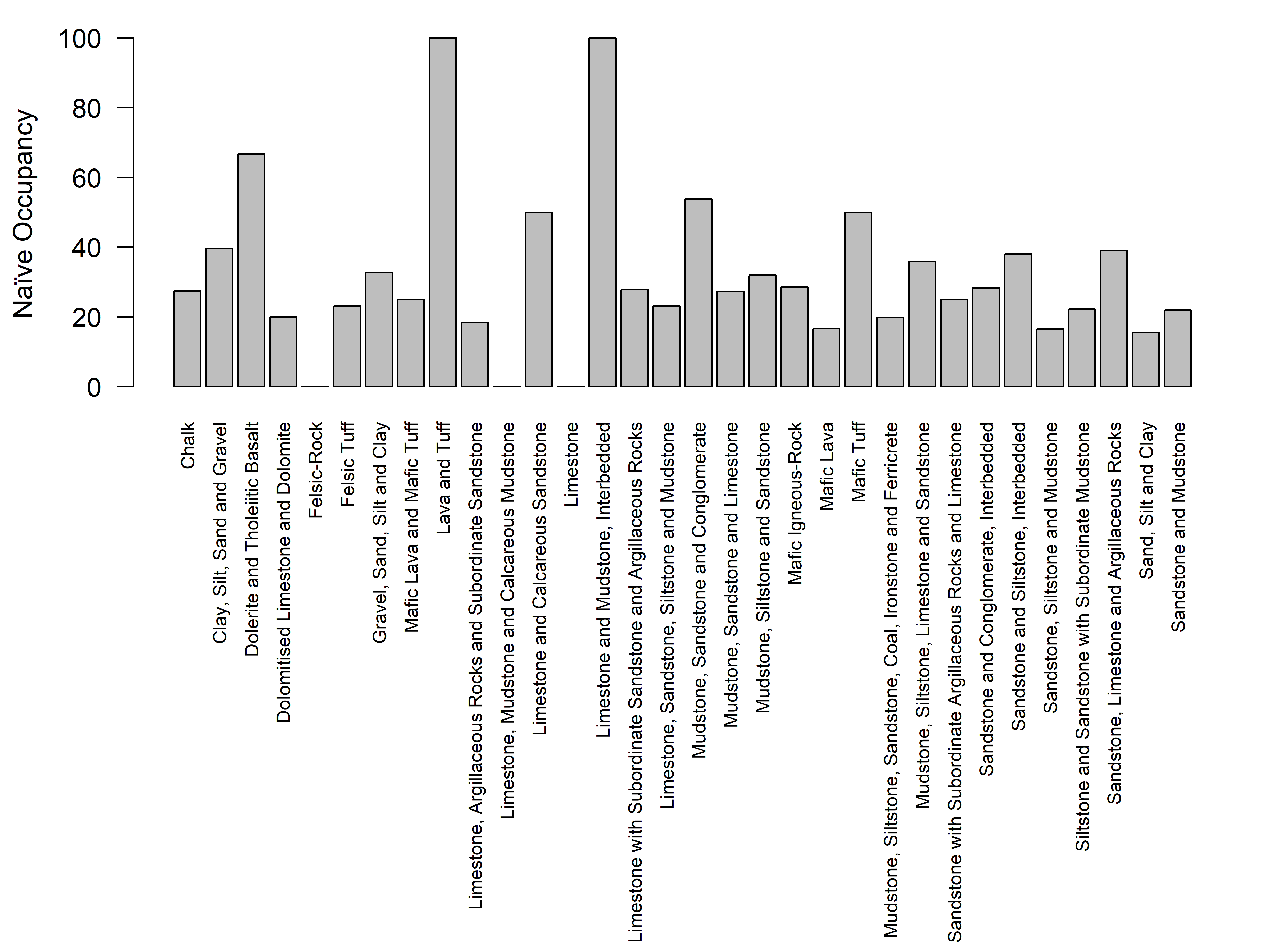


Figure S3 – Naïve great crested newt pond occupancy rate for each bedrock type


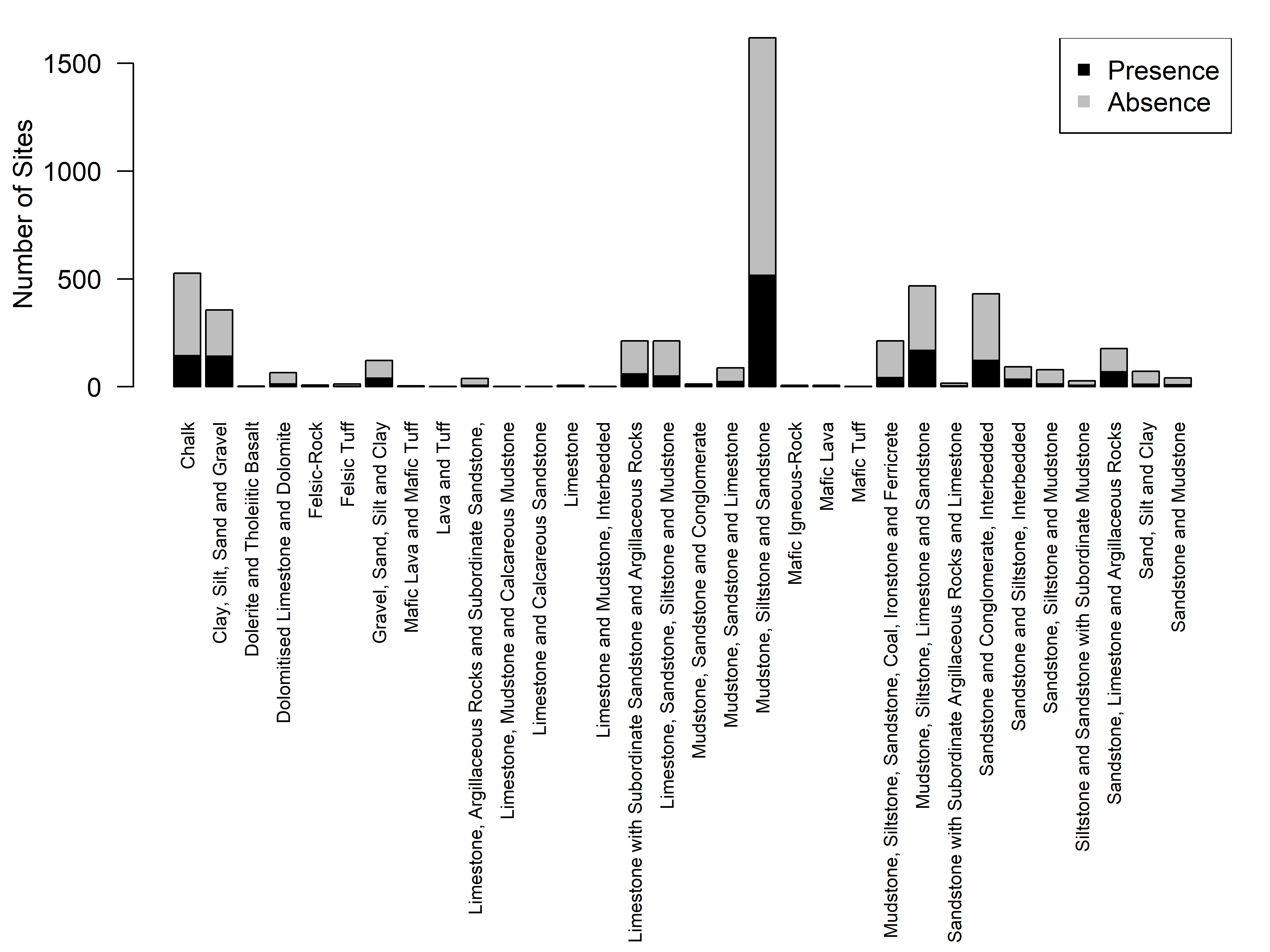


Figure S4 – the cumulative number of eDNA samples collected from each bedrock Type, with the great crested newt presence absence split shown


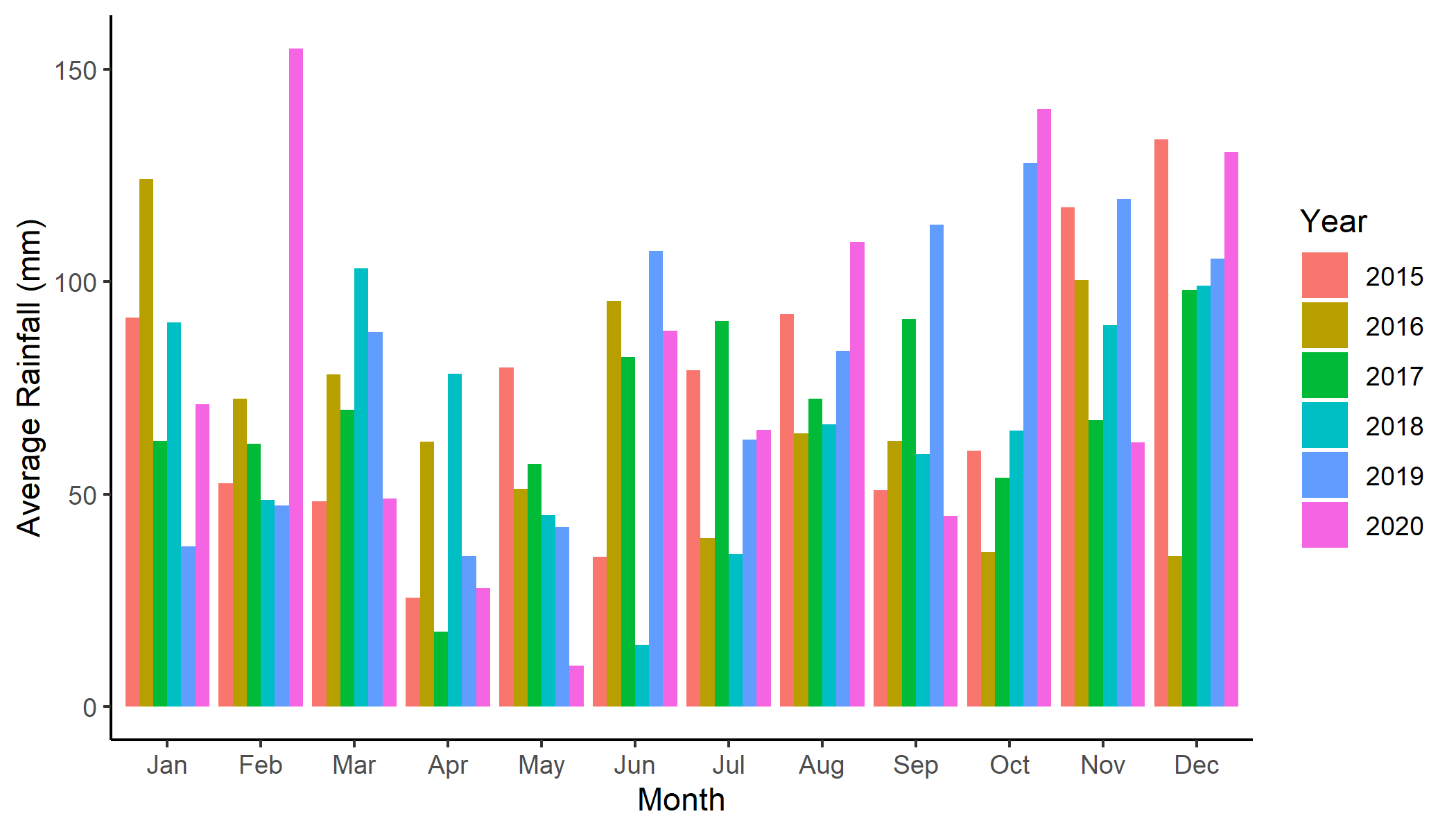


Figure S5 – Monthly average rainfall for England for the years 2015-2020 inclusive. Data extracted from Statista.com. <https://www.statista.com/statistics/610075/monthly-rainfall-in-england/>


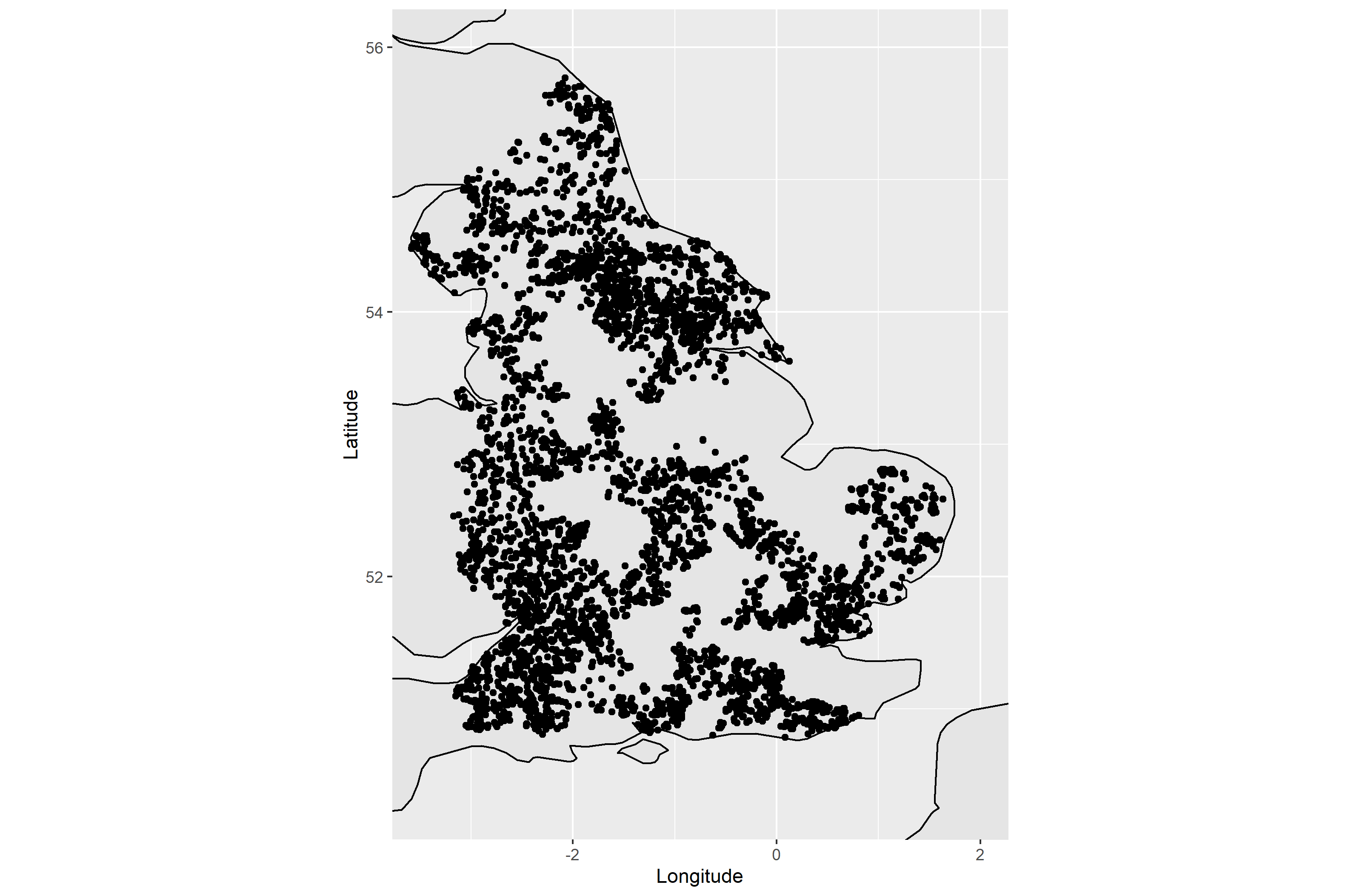


Figure S6 – Sample collection locations across much of England. Map created in R version 4.1.1^1^ using packages ‘ggplot2’ version 3.3.5^2^ and ‘maps’ version 3.4.0^3^.

**Supplementary Information References**

1. R Core Team. R: Language and Environment for Statistical Computing (2021).
2. Wickham H., Chang W., Henry L., Pedersen T.L., Takahashi K., Wilke C., Woo K., Yutani H., Dunnington D. Package ‘ggplot2’: Create Elegant Data Visualisations Using the Grammar of Graphics, R package version 3.3.5. (2021) <https://cran.r-project.org/web/packages/ggplot2/index.html>
3. Becker R. A., Wilks A.R., Brownrigg R., Minka T.P., Deckmyn A. Package ‘maps’: Draw Geographical Maps, R package version 3.4.0. (2021) <https://cran.r-project.org/web/packages/maps/index.html>
